# Draft genome sequence of *Desulfoplanes formicivorans* Pf12B^T^, a sulfate-reducing bacterium of the family *Desulfomicrobiaceae*

**DOI:** 10.1186/s40793-017-0246-2

**Published:** 2017-06-05

**Authors:** Miho Watanabe, Hisaya Kojima, Manabu Fukui

**Affiliations:** 1 The Institute of Low Temperature Science, Hokkaido University, Nishi 8, Kita 19, Kita-ku Sapporo, Hokkaido 060-0819 Japan; 2 Postdoctoral Research Fellow of the Japan Society for the Promotion of Science, Chiyoda-ku, Tokyo 102-8471 Japan

**Keywords:** *Bacteria*, Gram-negative, Anaerobe, Sulfate-reducer, *Desulfomicrobiaceae*

## Abstract

*Desulfoplanes formicivorans* strain Pf12B^T^ is the type strain of the type species in the genus *Desulfoplanes*, which is the one of the genera in the family *Desulfomicrobiaceae* within the order *Desulfovibrionales*. This deltaproteobacterium was isolated from a blackish meromictic lake sediment. *D. formicivorans* strain Pf12B^T^ is a Gram-negative, motile and sulfate-reducing bacterium. Cells of strain Pf12B^T^ are characterized by possession of vibroid morphology and red fluorescent pigment. Here we describe the features, draft genome sequence and annotation of this organism, the sole species of the genus *Desulfoplanes*. The genome comprised 3,000,979 bp, 2,657 protein-coding genes and 58 RNA genes.

## Introduction

Strain Pf12B^T^ (= NBRC 110391 ^T^ = DSM 28890 ^T^) is the type strain of *Desulfoplanes formicivorans*, which is the type species of the genus *Desulfoplanes* in the family *Desulfomicrobiaceae*. The family *Desulfomicrobiaceae* was proposed by Kuever et al. (2006) and contained only one genus, *Desulfomicrobium*. The genus *Desulfoplanes* was later added to this family because of the phylogenetic position [1]. All members of the family *Desulfomicrobiaceae* including *D. formicivorans* are sulfate reducers and incomplete oxidizers, which are unable to completely oxidize organic matters to CO_2_. All known strains of the genus *Desulfomicrobium* have rod- or ellipsoidal-shaped morphology and they all lack desulfoviridin, which is a red fluorescent pigment [2–4]. In contrast, *D. formicivorans* strain Pf12B^T^ was characterized by vibroid morphology and possession of red fluorescent pigment.

In this study we summarize the features of *D. formicivorans* strain Pf12B^T^ and provide an overview of the draft genome sequence and annotation of this strain.

## Organism Information

### Classification and features

**Fig. 1:**
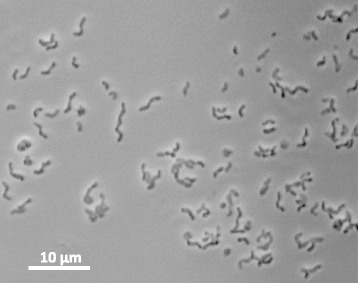
Photomicrograph of cells of *D. formicivorans* strain Pf12B^T^. Cells were grown with formate (10 mM) and yeast extract (0.5 g l^-1^) in the presence of sulfate for 2 days

*D. formicivorans* strain Pf12B^T^ was isolated from the anaerobic sediments of a meromictic lake [1, 5]. Cells of this strain are Gram-negative, motile, non-spore-forming and vibroids (Fig 1, Table 1). Under UV illumination, cell lysate of the strain exhibited red fluorescence suggesting the presence of desulfoviridin. Temperature range for growth is 13–50 °C, with an optimum temperature at 42–45 °C. NaCl concentration for growth is 0.5–8% (w/v) and optimal concentration is 1–4% (w/v). This bacterium is strictly anaerobic and is capable of respiration and fermentation. Sulfate, thiosulfate and sulfite are used as electron acceptors for growth. Nitrate is not used for respiration. Pyruvate, malate and fumarate are used for fermentative growth.

**Table 1:** Classification and general features of *Desulfoplaens formicivorans* strain Pf12B^T^ according to MIGS recommendations

MIGS ID	Property	Term	Evidence code^a^
	Classification	Domain *Bacteria*	TAS [6]
		Phylum *Proteobacteria*	TAS [18]
		Class *Deltaproteobacteria*	TAS [19, 20]
		Order *Desulfovibrionales*	TAS [20, 21]
		Family *Desulfomicrobiaceae*	TAS [4, 20]
		Genus *Desulfoplanes*	TAS [1]
		Species *Desulfoplanes formicivorans*	TAS [1]
		Type strain: Pf12B^T^ (DSM 28890)	
	Gram stain	negative	TAS [1]
	Cell shape	vibroid	TAS [1]
	Motility	motile	TAS [1]
	Sporulation	nonsporulating	TAS [1]
	Temperature range	13–50 °C	TAS [1]
	Optimum temperature	42–45 °C	TAS [1]
	pH range; Optimum	6.1–8.6; 7.0–7.5	TAS [1]
	Carbon source	organic acids	TAS [1]
MIGS-6	Habitat	Brackish meromictic lake sediment	TAS [1]
MIGS-6.3	Salinity	10–40 g NaCl /l	TAS [1]
MIGS-22	Oxygen requirement	obligate anaerobic	TAS [1]
MIGS-15	Biotic relationship	free-living	TAS [1]
MIGS-14	Pathogenicity	non-pathogen	NAS
MIGS-4	Geographic location	Kushiro, Hokkaido, Japan	TAS [1, 5]
MIGS-5	Sample collection	May 2012	TAS [5]
MIGS-4.1	Latitude	42° 58' 20.6" N	TAS [5]
MIGS-4.2	Longitude	144° 24' 6.6" E	TAS [5]
MIGS-4.4	Altitude	NA	

^a^Evidence codes - *TAS* Traceable Author Statement (i.e., a direct report exists in the literature), *NAS* Non-traceable Author Statement (i.e., not directly observed for the living, isolated sample, but based on a generally accepted property for the species, or anecdotal evidence). NA; not avairable.

**Fig. 2:**
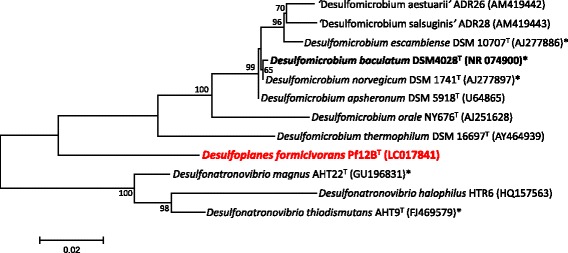
Phylogenetic tree showing the relationship of *D. formicivorans* strain Pf12B^T^ to other species of the family *Desulfomicrobiaceae*. Members of the genus *Desulfonatronovibrio* were used as the outgroup in this analysis. The tree was constructed by the Maximum-Likelihood method with MEGA version 5.1 [16] based on ClustalX version 2.1 [17] aligned sequences of 16S rRNA gene. Bootstrap values (percentages of 1000 replications) of ≥ 50% are shown at nodes. The presence of sequenced genome is indicated with superscripted “*”

Phylogenetic relationship of *D. formicivorans* strain Pf12B^T^ and all members of the family *Desulfomicrobiaceae* are shown in the 16S rRNA gene phylogenetic tree (Fig. 2). *D. formicivorans* strain Pf12B^T^ is assigned to the family *Desulfomicrobiaceae* but forms a well-separated branch among other cultivated relatives of the same family.

## Genome sequencing information

### Genome project history

*D. formicivorans* strain Pf12B^T^ was selected for genome sequencing on the basis of its 16S rRNA gene-based phylogenetic position in the family *Desulfomicrobiaceae* (Fig. 2). A summary of the genome sequencing project information and its association with MIGS version 2.0 compliance [6] are shown in Table 2. The genome consists of 26 contigs, which has been deposited at DDBJ/EMBL/GenBank under accession number BDFE00000000.

**Table 2:** Project information

MIGS ID	Property	Term
MIGS 31	Finishing quality	High-quality draft
MIGS-28	Libraries used	TruSeq Nano DNA library prep kit
MIGS 29	Sequencing platforms	Illumina Hiseq paired-end
MIGS 31.2	Fold coverage	370×
MIGS 30	Assemblers	Velvet version 1.2.08
MIGS 32	Gene calling method	Microbial Genome Annotation Pipeline (MiGAP)
	Locus Tag	BDFE01000001-BDFE01000026
	Genbank ID	BDFE00000000
	GenBank Date of Release	June 30, 2016
	BIOPROJECT	PRJDB4875
MIGS 13	Source Material Identifier	DSM 28890
	Project relevance	Ecology and evolution

### Growth conditions and genomic DNA preparation

*D. formicivorans* strain Pf12B^T^ (DSM 28890) was grown on bicarbonate-buffered sulfide-reduced medium [7] containing 28 mM sulfate, 10 mM formate and 0.5 g l^-1^ yeast extract at 45 °C. Genomic DNA was extracted from collected cells using Wizard® genomic DNA purification kit (Promega).

### Genome sequencing and assembly

The genome of strain Pf12B^T^ was sequenced using paired-end Illumina sequencing at Hokkaido System Science Co., Ltd. (Japan). From a library with 350 bp inserts, the 10,511,386 reads were generated. After trimming of the reads, a total of 9,393,309 high-quality filtered paired end reads with a hash length of 95 bp were obtained. Reads were assembled *de novo* using Velvet version 1.2.08 into 26 high quality scaffolds. Gap closing analysis in these scaffolds was performed using Platanus version 1.2.1.

### Genome annotation

Draft genome sequences were automatically annotated using the MiGAP [8]. In the pipeline, RNAmmer [9] and tRNAscan-SE [10] were used to identify rRNA and tRNA genes, respectively. MetaGene Annotator [11] was used to predict ORFs likely to encode proteins (CDSs), and functional annotation was performed based on reference databases, including RefSeq, TrEMBL, and COGs. Manual annotation was performed using IMC-GE software (In Silico Biology; Yokohama, Japan). Putative CDSs were confirmed again by a sequence similarity search using the BLASTP tool. Putative CDSs possessing BLASTP matches with more than 70% coverage and 35% identity and E-values less than 1 × e^−5^ were considered potentially functional genes. When these cut-off values were not satisfied, the CDSs were annotated as hypothetical proteins. Transcription start sites of predicted proteins were corrected based on multiple sequence alignments. If the distance between CDSs was larger than 500 bp, further ORF extraction for coding genes was performed.

The protein-coding genes in the genome were also subjected to analysis on WebMGA [12] for the COGs and Protein family (Pfam) annotations. Transmembrane helices and signal peptide prediction were analyzed using Phobius [13]. CRISPR loci were distinguished using the CRISPR Recognition Tool [14].

## Genome properties

The total genome of strain *D. formicivorans* strain Pf12B^T^ was 3,000,979 bp in size with a GC content of 49.81% (Table 3). It was predicted to contain 2,715 genes including 2,657 protein-coding genes and 58 RNA genes (for tRNA and rRNA). Approximately 83% of the predicted genes were assigned to COG functional categories. The distribution of genes into COGs functional categories is presented in Table 4.

**Table 3:** Genome statistics

Attribute	Value	% of Total
Genome size (bp)	3,000,979	100.00
DNA coding (bp)	2,596,072	86.51
DNA G + C (bp)	1,494,788	49.81
DNA scaffolds	26	-
Total genes	2,715	100.00
Protein coding genes	2,657	97.86
RNA genes	58	2.14
Pseudo genes	NA	NA
Genes in internal clusters	NA	NA
Genes with function prediction	1888	69.54
Genes assigned to COGs	2255	84.87
Genes with Pfam domains	2110	79.41
Genes with signal peptides	356	13.40
Genes with transmembrane helices	570	21.45
CRISPR repeats	2	0.07

NA, not avairable

**Table 4:** Number of genes associated with general COG functional categories

Code	Value	%age	Description
J	156	5.75	Translation, ribosomal structure and biogenesis
A	0	0.00	RNA processing and modification
K	102	3.76	Transcription
L	110	4.05	Replication, recombination and repair
B	1	0.04	Chromatin structure and dynamics
D	29	1.07	Cell cycle control, Cell division, chromosome partitioning
V	25	0.92	Defense mechanisms
T	210	7.74	Signal transduction mechanisms
M	169	6.23	Cell wall/membrane biogenesis
N	105	3.87	Cell motility
U	93	3.43	Intracellular trafficking and secretion
O	110	4.05	Posttranslational modification, protein turnover, chaperones
C	222	8.18	Energy production and conversion
G	116	4.27	Carbohydrate transport and metabolism
E	234	8.62	Amino acid transport and metabolism
F	65	2.39	Nucleotide transport and metabolism
H	101	3.72	Coenzyme transport and metabolism
I	51	1.88	Lipid transport and metabolism
P	122	4.50	Inorganic ion transport and metabolism
Q	37	1.36	Secondary metabolites biosynthesis, transport and catabolism
R	258	9.51	General function prediction only
S	168	6.19	Function unknown
-	459	16.91	Not in COGs

## Insights from the genome sequence

**Fig. 3:**
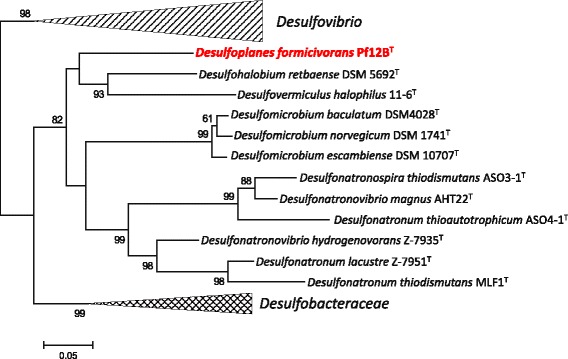
Phylogenetic tree based on DsrAB amino acid sequence of *D. formicivorans* strain Pf12B^T^ and members of the orders *Desulfovibrionales* and *Desulfobacterales*. The tree was constructed by the Maximum-Likelihood method with MEGA version 5.1 [16] based on ClustalX version 2.1 [17] aligned protein sequences. Bootstrap values (percentages of 1000 replications) of ≥ 50% are shown at nodes

The draft genome provides interesting phylogenetic and metabolic information, including phylogeny of *dsr* genes, which are essential for dissimilatory sulfate reduction. The *dsrAB* genes are frequently used as marker genes to evaluate phylogenetic relationship of sulfate-reducing bacteria, as well as to reveal their diversity and distribution in environments. Phylogenetic analysis based on DsrAB amino acid sequence was performed to disclose the phylogenetic position of *D. formicivorans* strain Pf12B^T^ among sulfate reducers belonging to the families *Desulfovibrionales* and *Desulfobacterales* (Fig. 3). In the resulting phylogenetic tree, *D. formicivorans* strain Pf12B^T^ was clearly separated from all members of the family *Desulfomicrobiaceae*. This result partially conflicts with the 16S rRNA gene phylogeny, and this contradiction may represent a new case of lateral gene transfer event which frequently has been found among dissimilatory sulfate-reducing and sulfur-oxidizing bacteria [15].

## Conclusions

Draft genome sequence of *D. formicivorans* strain Pf12B^T^ described here is the first published genome sequence of a member of the genus *Desulfoplanes*, which is a newly proposed taxon in the family *Desulfomicrobiaceae*. The genome of the strain Pf12B^T^ consists of 2,657 protein-coding genes and 58 RNA genes. DsrAB phylogenetic tree shows the strain Pf12B^T^ is located in the independent position, which is distant from a cluster of *Desulfomicrobium* species.

## Abbreviations

CRISPR: Clustered regularly interspaced short palindromic repeat
Dsr: Dissimilatory sulfite reductase
MiGAP: Microbial Genome Annotation Pipeline
